# Overexpression of *BnaAOX1b* Confers Tolerance to Osmotic and Salt Stress in Rapeseed

**DOI:** 10.1534/g3.119.400366

**Published:** 2019-09-04

**Authors:** Hongli Yang, Linbin Deng, Hongfang Liu, Shihang Fan, Wei Hua, Jing Liu

**Affiliations:** Oil Crops Research Institute of the Chinese Academy of Agricultural Sciences, Key Laboratory of Biology and Genetic Improvement of Oil Crops, Ministry of Agriculture and Rural Affairs, Wuhan 430062, P.R. China

**Keywords:** AOX1b, osmotic stress, salt stress, abscisic acid, rapeseed

## Abstract

Alternative oxidases (AOXs) are the terminal oxidase in the cyanide-resistant respiration pathway in plant mitochondria, which play an important role in abiotic stress and are proposed as a functional marker for high tolerant breeding. In this study, ten AOX genes (*BnaAOXs*) were identified, and CysI and CysII of AOX isoforms were highly conserved in rapeseed. Among them, *Bna.AOX1b* was mainly expressed in the ovule and displayed varying expression between rapeseed cultivars which showed different salt resistance in seed germination. We identified its mitochondrial localization of this gene. To investigate the function of *BnaAOX1b* in rapeseed, transgenic rapeseed lines with overexpressed *BnaAOX1b* were created and seed germination and seedling establishment assays were performed under osmotic, salt, and ABA treatment. The results indicated that overexpression of *BnaAOX1b* significantly improved seed germination under osmotic and salt stress and weakened ABA sensitivity. In addition, post-germination seedling growth was improved under high salt condition, but showed hypersensitivity to ABA. RNA-sequencing analysis indicated that the genes involved in electron transport or energy pathway were induced and a number of gene responses to salt stress and ABA were regulated in *Bna.AOX1b* overexpressing seeds. Taken together, our results imply that *Bna.AOX1b* confers tolerance to osmotic and salt stress in terms of seed germination and seedling establishment by regulating stress responsive genes and the response to ABA, and could be utilized as a candidate gene in transgenic breeding.

Seeds are important for sustainable agriculture production and seed germination is a complex process affected by many factors. High salinity soil inhibits seed germination, and high capacity seed germination under salinity stress is an important objective of crop breeding (He *et al.* 2019; Wang *et al.* 2011). Seed germination recovery is an adaptive trait for the successful establishment and dispersal of extremophile plants in their native ecosystems. Accumulating evidence indicates that reactive oxygen species (ROS) are key regulators in seed physiology and mediate seed germination (Bailly 2004; Bailly *et al.* 2008; Bouteau Hel *et al.* 2007; Ye *et al.* 2012). Disruption of ROS homeostasis reduces the ability of seed germination. Alternative respiratory pathway plays important roles in temperature, nutrient, heavy metal, high light, drought and salt stress by minimizing the generation of ROS and allowing a degree of metabolic flexibility in the cell (Vanlerberghe 2013; Vishwakarma *et al.* 2015). The alternative respiratory pathway mediated by the NAD(P)H dehydrogenase and alternative oxidase (AOXs) in plant mitochondria plays an important role in balancing the ATP/ NAD(P)H ratio and maintaining redox homeostasis (Vanlerberghe 2013; Rasmusson *et al.* 2004). AOX are the terminal oxidases of plant mitochondria in their respiratory electron transport chain and the AOX pathway diverts electrons from the ubiquinone pool and reduces oxygen to water with neither proton translocation nor ATP synthesis (Affourtit *et al.* 2002).

In higher plants, AOX proteins are encoded by two discrete gene subfamilies, AOX1 and AOX2. *AOX1* genes are present in both monocot and eudicot plant species, whereas the *Aox2* genes are mainly found in eudicot species (Considine *et al.* 2002). The gene expression of these isoforms is varied, depending on tissues, growth, development, and the environment (Wellmer *et al.* 2004; Clifton *et al.* 2006; Hennig *et al.* 2004; Sweetman *et al.* 2018). It has been demonstrated that the AOX pathway is critical for resistance to biotic and abiotic stress conditions (Zhang *et al.* 2009; Arnholdt-Schmitt *et al.* 2006; Fiorani *et al.* 2005; Giraud *et al.* 2008; Zhang *et al.* 2017; Murakami and Toriyama 2008). In Arabidopsis and rice, *AOX1a* and *AOX1d* are the most stress responsive genes to abiotic stress including salinity, drought, low temperature and high temperature (Saika *et al.* 2002; Ito *et al.* 1997; Li *et al.* 2013; Clifton *et al.* 2006). *AOX* not only allows Arabidopsis to germinate at low temperature, but also can prevent the generation of peroxides under adverse conditions, to avoid adverse effect on various cellular functions (Fiorani *et al.* 2005). The absence of *AOX1a* in Arabidopsis results in acute sensitivity to combined light and drought stress (Giraud *et al.* 2008). Overexpression of wheat *AOX1a* alters respiration capacity and response to ROS under low temperature in transgenic Arabidopsis (Sugie *et al.* 2006). In tobacco, the signaling molecules salicylic acid (SA), nitric oxide (NO), H_2_O_2_ are known to induce the stress responsive *AOX* genes (Zhang *et al.* 2009).

In addition to transcriptional regulation, it has been shown that AOX activity is post translationally regulated. AOX is a di-iron carboxylate protein and is activated by redox mechanisms and 2-oxo acids (Selinski *et al.* 2016; Siedow and Umbach 2000). In the N-terminal domain of the AOX protein, most isoforms possess two highly conserved Cys residues (CysI and CysII), which are both involved in 2-oxo acid activation. Near the catalytic di-iron center, minority isoforms such as AOX1a possess a third Cys residue (CysIII) that is not involved in activation by reduction or metabolites, but substitutions at this position affect activity (Selinski *et al.* 2017).

Rapeseed is one of the most important sources of edible vegetable oil and forage protein, as well as the most important raw material for biodiesel. Various abiotic stresses, such as salinity, heat, drought, and cold are responsible for major yield loss. Previous studies showed that *AOX1* genes are important to stress tolerance and yield stability. Little is known about the role of *AOX1b* genes in plant. In this study, transgenic rapeseed lines of overexpressing *BnaAOX1b* were generated by Agrobacterium-mediated transformation. Our data indicate that *BnaAOX1b* improved the seed germination rate and seedling establishment under osmotic or salt stress, which represents a potential application of this gene in transgenic breeding.

## Materials and methods

### Quantitative real-time PCR (qRT-PCR) analysis

Total RNA from developing ovule (DAF 15-45) was extracted for quantitative RT-PCR. RNA preparations were treated with DNase to remove contaminating DNA, and RNA (1 µg) was subjected to reverse transcription using the First Strand cDNA Synthesis Kit for RT-PCR (Takara, Japan). cDNA products were diluted 10-fold prior to use and the quantitative real-time PCR was carried out using a ABI 7500 System (ABI). System software by the comparative ⊿⊿cycle threshold method was used for quantification. The specificity of the amplification for each primer pair was verified by melting curve analysis and the expression level of target gene was normalized to the amount of *TMA7*, which has a highly stable expression level in different tissues and under various growth conditions. Primers used in the qPCR were listed in Table S1.

### Expression patterns of BnaAOX1b genes in different tissues of rapeseed

For expression profiling of *BnaAOX* genes, we used several groups of RNA-seq data obtained earlier by our group. The transcript abundance was calculated by reads per kilobase per million mapped reads (RPKM, values were log2) (Audic and Claverie 1997). Hierarchical clustering analysis of the transcriptional profiles was performed using the hclust command in R.

### Protein localization

The full-length CDS of *Bna*.*AOX1b* without the termination codon (TGA) was PCR-amplified using the *Bna*.*AOX1b*-GFP-F and *Bna1.AOX1b*-GFP-R primers (Table S1) and inserted into the pM999-GFP vector. The BnaAOX-GFP fusion construct was introduced into rapeseed protoplasts by PEG/calcium-mediated transformation. The commercially available mitochondrial fluorescent probe (MitoTracker Red CMXRos, 50 nM) was used as an indicator of the mitochondrion. Fluorescence signals were detected and photographed using a confocal laser microscope.

### Plasmid constructs and generation of BnAOX1b transgenic lines of rapeseed

The coding region of *AOX1b* was amplified from ZS11 with the primers designed against the rapeseed cDNA sequences using KOD plus neo (Toyobo Taq DNA polymerase, Japan). PCR amplifications were carried out with 35 cycles of 98° for 10 s, 55° for 30 s, and 68° for 30 s. The amplicons were introduced into Gateway entry vector pCR/GW/TOPO (Invitrogen, USA) and then recombined into pEarleyGate100 using the Gateway LR Clonase Enzyme Mix (Invitrogen, USA).

The construct was transferred into *Agrobacterium tumefaciens* strain GV3101 and transformed into the rapeseed line ZS11 by hypocotyls transgenic system. Transgenic plants were selected using 200μg/ml Basta (Sigma, USA) and confirmed by PCR as expression vector containing the 35S promoter primer Gate-35S and *BnaAOX1b-R*. 18 T_1_ independent *35S*::*BnAOX1b* transformants were gotten and grown up to T3 generation. Homozygous plants were identified and detected the transcript abundance.

### Osmotic, salt and ABA tolerance assay

For seed germination assay, seeds were surface sterilized and sown on 1/2MS medium (agar 0.8%, sucrose 3%); 1/2MS medium containing different concentrations of NaCl (100, 150 and 200 mM), mannitol (300mM) and ABA (10 μM ABA). The germination rate was calculated based on radicle protrusion. To study the effect of salt stress on seedling growth, the roots of seedlings (DAS7) were immersed into nutrient solution supplemented with 200 mM Nacl. Seven days later, fresh weight of transgenic and control plants were measured. For seedling growth assay under ABA treatment, seeds with radicle protrusion grown on 1/2 MS plates for 3 days were transferred vertically to 1/2 MS plates supplemented with 10–20 μM ABA. The seedlings grown on 1/2 MS plates without stress were employed as the control. Root lengths of seedlings were measured after 7 d of upright growth in medium.

### Transcriptome analyses of Bna.AOX1b-regulated pathways

For each gene, the expression level was measured by fragments Per Kilobase exon Model per Million mapped reads (FPKM) based on the number of uniquely mapped reads, to eliminate the influence of different gene lengths and sequencing discrepancies on the gene expression calculation. Differential expression was performed with the DESeq2 R package using [log2 (Fold change)]≥ 1 and a corrected *P* < 0.05 as the threshold for significant differential expression (Love *et al.* 2014). P-values were adjusted using the Benjamini and Hochberg’s approach for controlling the false discovery rate. Functional enrichment analysis including Gene Ontology (GO) and KEGG were performed on DEGs, which were compared to the whole genome background using hypergeometric test with Benjamini and Hochberg false discovery rate correction at the significance threshold of 0.05.

### Data availability

Strains and plasmids are available upon request. The authors affirm that all data necessary for confirming the conclusions of the article are present within the article, figures, tables and supplemental material. Supplemental material available at FigShare: https://doi.org/10.25387/g3.9759698.

## Results

### Identification and sequence analysis of the rapeseed AOX genes

**Figure 1:**
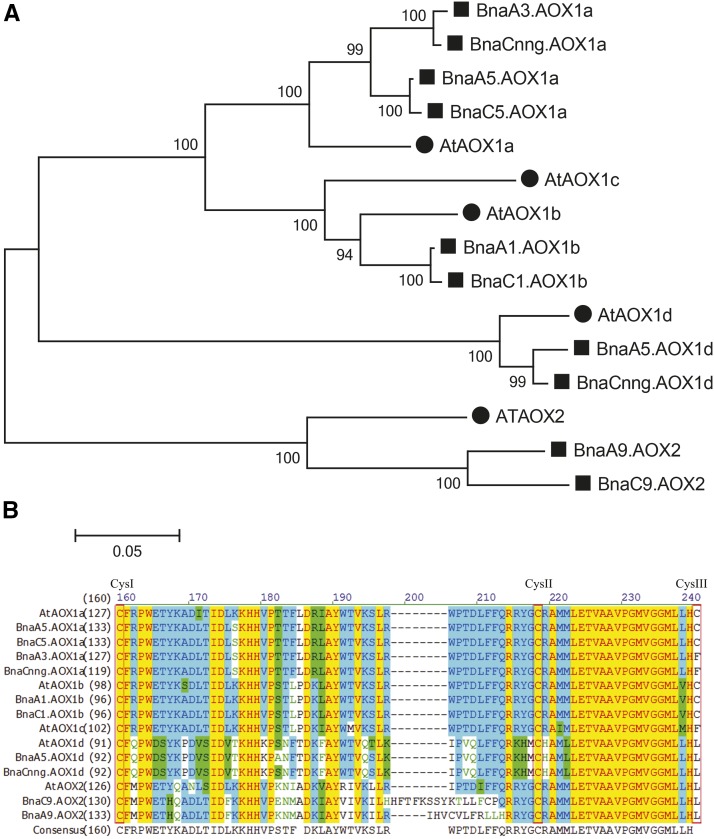
Phylogenetic and sequence analysis of BnaAOX genes. a, The phylogenetic tree was constructed using the neighbor-joining (NJ) method in MEGA6.0 software; b, Structural analysis of *BnaAOX* genes. Lengths of exons (yellow) and introns (black) of each *BnaAOXs* are displayed proportionally; c, The protein sequences of AOX from rapeseed and Arabidopsis were aligned using Vector NTI software.

AOX genes belong to a small gene family and homologs share high sequence identities. In this study, eight AOX1-type genes and two AOX2-type genes were identified by whole genome bioinformatics analyses. Phylogenetic analysis was conducted to assess the relationship between rapeseed and Arabidopsis. The rapeseed AOX genes were renamed to comply with the nomenclature of their closest Arabidopsis homologs and chromosomal localization. No rapeseed ortholog corresponding to AOX1c-type gene was identified (Figure 1a). The size of the deduced AOX proteins varied greatly, ranging from 319 (BnaC5.AOX1d) amino acids to 504 amino acids (BnaC9.AOX2, Table 1). BnaA5.AOX1d and BnaCnng.AOX1d with their Arabidopsis homology exhibited the highest sequence similarity (>90%, Table1). The majority of BnaAOX genes contained 4 exons whereas BnaC9.AOX2 displayed more volatile structures and contained nine exons (Table1). Sequence analysis suggested that rapeseed contained two BnaAOX1b homologs, which located in A1 chromosome and C1 chromosome, respectively. We designed PCR primers to amplify the entire coding region sequences of *BnaA1.AOX1b* and *BnaC1.AOX1b* which consisted of 972bp and 975bp, respectively. The predicted polypeptides encoded by *BnaAOX1b* were homologous to AtAOX1b (At3g22360), with 87.8% and 87.6% identity, respectively.

**Table 1:** The AOX genes identified in rapeseed genome

Gene name	Gene ID	Arabidopsis ortholog.	Identity (%)	Contig Location	strand	Exon no.	Transcript length	Translation length
BnaA5.AOX1a	BnaA05g17260D	AT3G22370	85.83	LK032005:166,198-168,192	+	4	1516	360
BnaC5.AOX1a	BnaC05g30210D	AT3G22370	84.97	LK032399:331845-333746	+	4	1425	360
BnaA3.AOX1a	BnaA03g36620D	AT3G22370	79.78	LK031874:183355-187360	+	6	1358	354
BnaCnng.AOX1a	BnaCnng63430D	AT3G22370	82.32	LK039388:931-3325	—	4	1319	346
BnaA1.AOX1b	BnaA01g24740D	AT3G22360	86.9	LK032331:412578-414387	—	4	972	323
BnaC1.AOX1b	BnaC01g31620D	AT3G22360	86.9	LK032661:147,775-149,414	—	4	972	323
BnaA5.AOX1d	BnaA05g35970D	AT1G32350	93.1	LK033183: 46,617-50,203	+	4	1314	319
BnaCnng.AOX1d	BnaCnng60000D	AT1G32350	91.54	LK037757:2452-4441	+	4	1216	319
BnaA9.AOX2	BnaA09g06750D	AT5G64210	77.96	LK032087:696154-698097	+	6	1156	363
BnaC9.AOX2	BnaC09g06270D	AT5G64210	76.99	LK031824:147485-151646	—	9	1515	504

Conserved Cys residues are involved in 2-oxo acid activation and affects basal activities of AOX isoforms. The regions that included all Cys residues of rapeseed and Arabidopsis AOX isoproteins were aligned using the bioinformatic tools Clustal W. The results indicated that CysI and CysII of rapeseed AOX isoforms are highly conservatived, whereas the loci of CysIII was Phe for two AOX1a alleles (BnaA3.AOX1a and BnaCnng.AOX1a) and Leu for rapeseed AOX1d and AOX2 isoforms (Figure 1b).

### BnaAOX1b differential expression in 51070 and zy036

**Figure 2:**
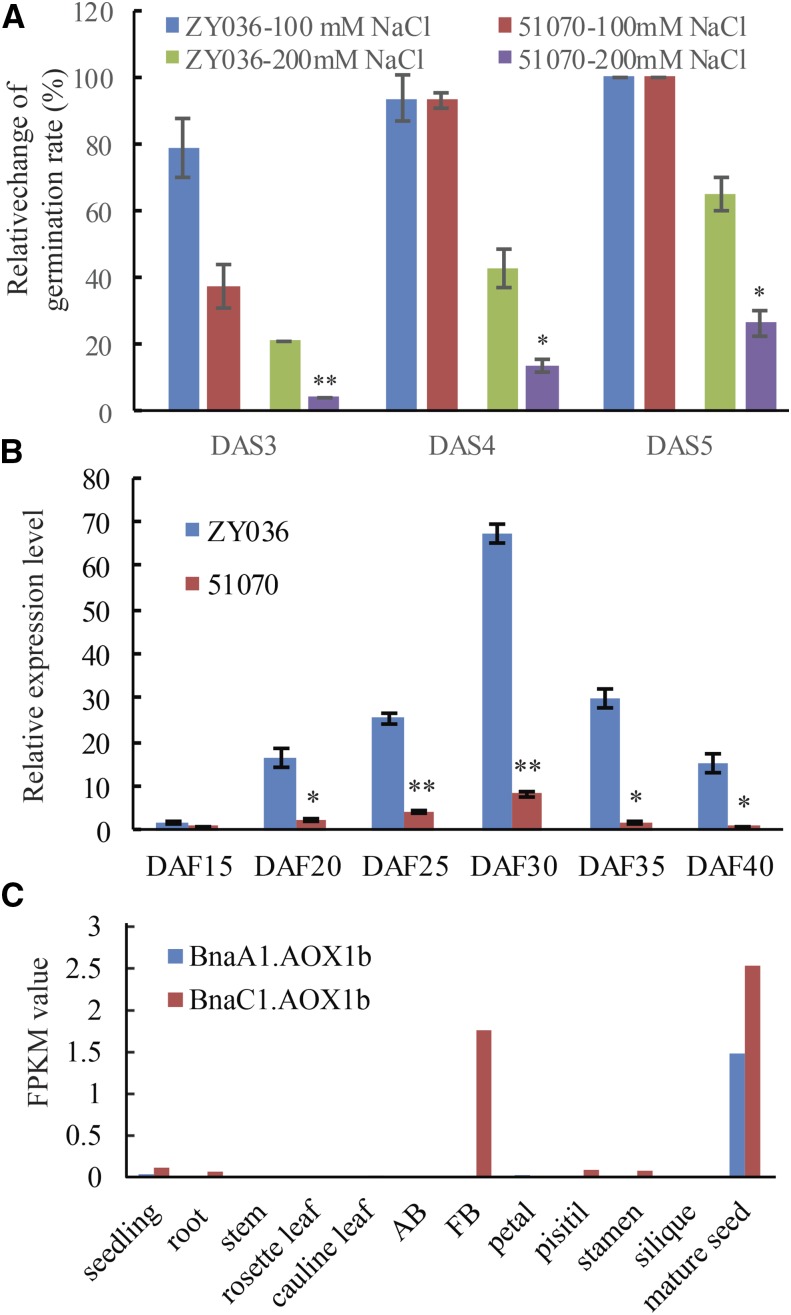
The expression pattern of BnaAOX1b in rapeseed. a, Germination rate of zy036 and 51070 under salt stress (% relative to control group); b, Expression analyses of *BnaAOX1b* in developing seed between rapeseed lines zy036 and 51070; c, The tempo-spatial transcription profiles of BnaAOX genes in various tissues.

The rapeseed cultivar zy036 exhibited a faster germination rate than cultivar 51070 under salt stress (Figure 2a). The germination rate of 51070 under 200 mM NaCl was only 2.78% of the control group at DAS 3. 51070 decreased by 75 percentage points and the rate of zy036 decreased only 35 points at DAS 5. Gene expression analyses of developing ovules from zy036 and 51070 revealed differential expression of *Bna.AOX1a* and *Bna.AOX1b*. The transcriptional abundance of *Bna.AOX1b* from two cultivars first increased then decreased with ovule development. The highest transcriptional abundance occurred at 30 days after flowering (DAF) with the expression level of *Bna.AOX1b* >eightfold higher in zy036 than in 51070. The greatest difference in expression level occurred at DAF 35 and the expression level of *Bna.AOX1b* was >18-fold higher in zy036 than in 51070 (Figure 2b). The expression level of gene *Bna.AOX1a* in zy036 was significantly higher than that in 51070 from 20 days after flowering (Fig. S1a, b).

To investigate the *Bna.AOX1* expression pattern in detail, transcript levels of *Bna.AOX1a* and *Bna.AOX1b* were analyzed in different rapeseed tissues including seedling, root, stem, rosette leaf, cauline leaf, axillary bud, flower bud, petal, pistil, stamen, silique and mature seed. *BnaC1.AOX1b* exhibited relatively high expression levels in mature seed and flower bud, and low expression level in seedling, root, cauline leaf, pistil and stamen. The transcripts of *BnaA1*.*AOX1b* were barely detectable in most tissues with the exception of mature seed (Figure 2c). *Bna.AOX1a* genes at four loci showed high transcriptional abundance in most tissues except mature seeds (Fig.S1). *AOX1a* gene has been studied deeply, while *Bna.AOX1b* gene is rarely reported. *BnaC1.AOX1b* was the dominant expressed gene compared with *BnaA1.AOX1b* and should be the focus of further research.

### BnaAOX1b is localized in the mitochondrial

**Figure 3:**
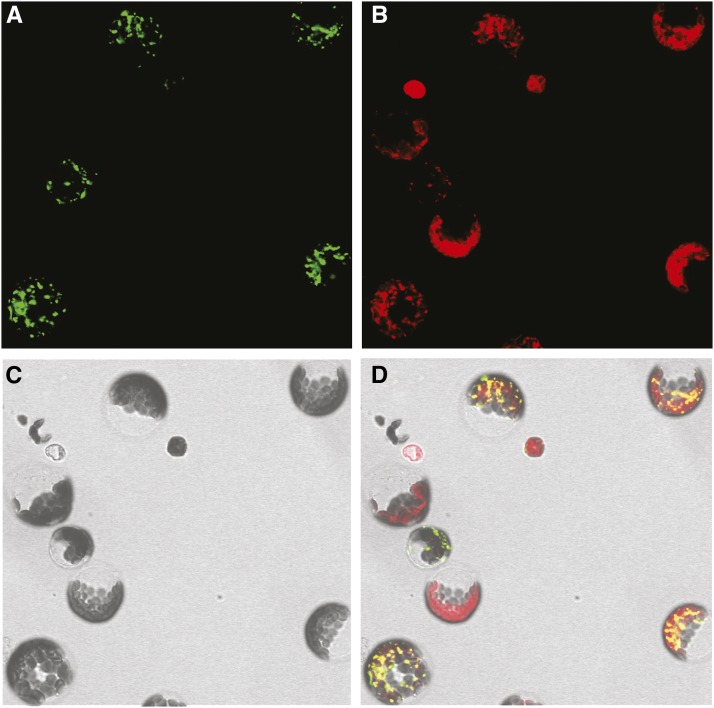
The subcellular localization of Bna.AOX1b. a, The protoplast showed a green fluorescent signal after transformation with the GFP-BnaAOX1b fusion proteins; b, The commercially available mitochondrial fluorescent probe (MitoTracker Red CMXRos, 50 nM) was used as an indicator of the mitochondrion; c, The same protoplast as (a) under bright field. d, The images in (a), (b) and (c) were merged. Bars = 10 μm in a-d.

To verify the subcellular localization of *BnaAOX1b* (C1 loci for functional analysis), its full length protein, without the stop code, was fused with green fluorescent protein (GFP) and driven by a constitutive Cauliflower mosaic virus (CaMV) 35S promoter. The fused construct was introduced into rapeseed protoplasts by PEG/calcium-mediated transformation. The transformed protoplasts were treated with MitoTracker Red CMXRos (50 nM), a commercially available mitochondrial fluorescent probe, which visualized the location of active mitochondria in the cytoplasm of these living cells. By co-staining with MitoTracker Red CMXRos and overlaying with bright field images, we found that the green fluorescence is retained in the mitochondria (Figure 3). This result is consistent with the presumed function of AOX as a terminal oxidase of the cyanide-resistant respiratory pathway in the plant mitochondrial respiratory chain.

### Overexpression of Bna.AOX1b in rapeseed improves seed germination under osmotic stress

**Figure 4:**
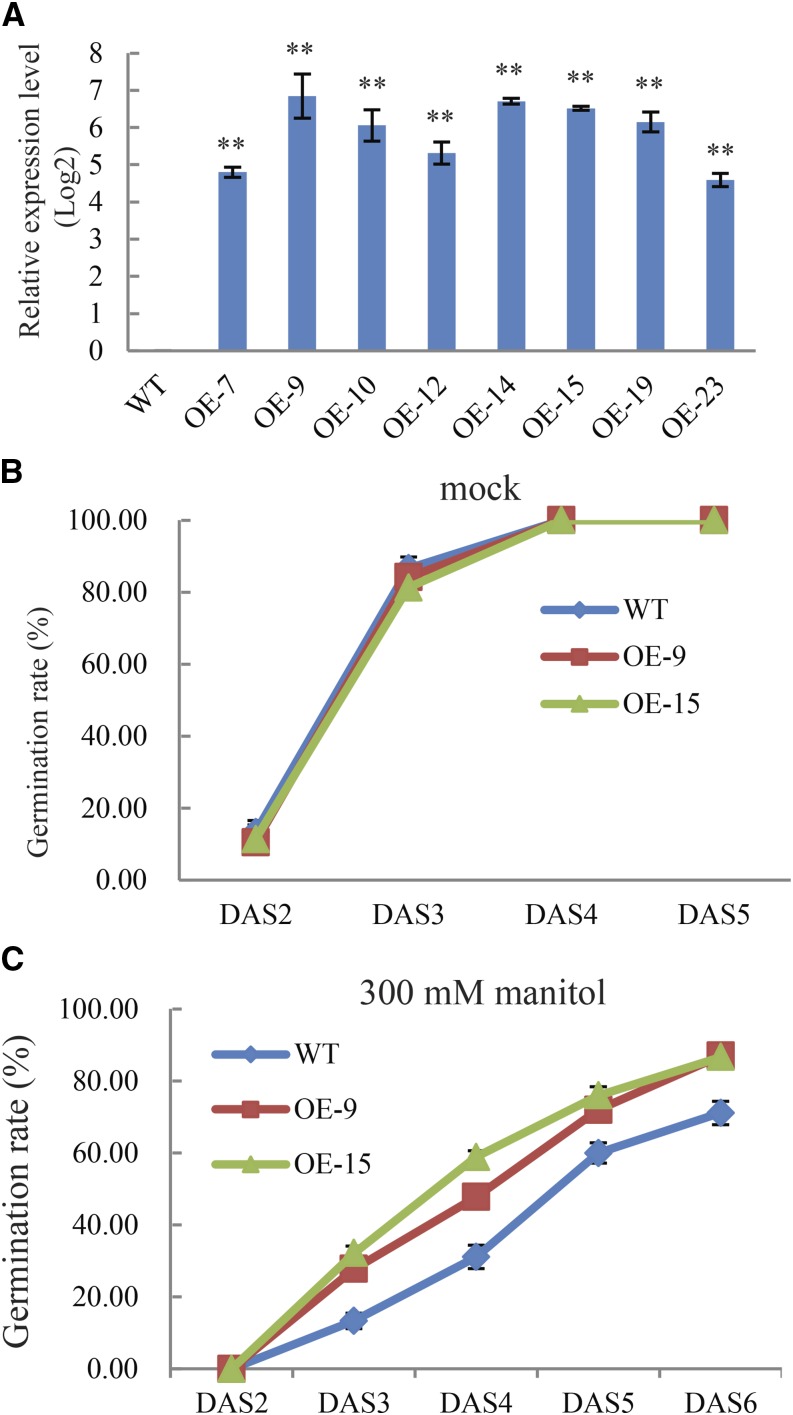
Characterization of transgenic *BnaAOX1b* rapeseed plants and comparison of germination rates between transgenic lines and wild type plants (WT) under osmotic stress. a, Expression level of *BnaAOX1b* in WT and OE lines. Data expression was normalized to and relative to the expression of *TMA7*. Data presented are mean values of three biological replicates and error bars represent standard deviations; b, Germination rates under normal condition. The seeds of WT and transgenic plants seeds of T_3_ generation were planted on 1/2MS agar medium; c, Germination rates under osmotic stress. The seeds of WT and transgenic plants seeds of T_3_ generation were planted on 1/2MS agar medium containing 300 mM manitol. Values shown are means ± SE (n = 3).

To reveal the *in vivo* role of rapeseed *AOX1b*, we constructed *Bna*.*AOX1b*-overexpressing transformations in the rapeseed cultivar ZS11. After selection by Basta resistance, the expression levels of *Bna.AOX1b* in the T1 generation of *35S*:: *Bna.AOX1b* transgenic plants were verified by real time PCR. The results indicated that *Bna.AOX1b* was highly expressed in all the selected transgenic lines and no transcript was found in WT leaves (Figure 4a). Transgenic rapeseed plants that overexpressed *Bna*.*AOX1b* were studied to determine if the overexpression of *Bna*.*AOX1b* could enhance stress tolerance.

To verify the effect of water deficiency on seed germination, seed germination assays were performed on 1/2 MS medium supplemented with 300 mM mannitol. The results showed that both WT and OE lines initiated germination similar to untreated controls at DAS 3. However, the germination rate of WT decreased by 28%, where the rate of transgenic lines decreased only by 6–13%. At DAS 6, the germination rate of OE lines was not different between stress treatment and normal conditions whereas seed germination of WT was substantially inhibited (86.67–71.11%, Figure 4b-c).

### Bna.AOX1b enhances salt stress tolerance

**Figure 5:**
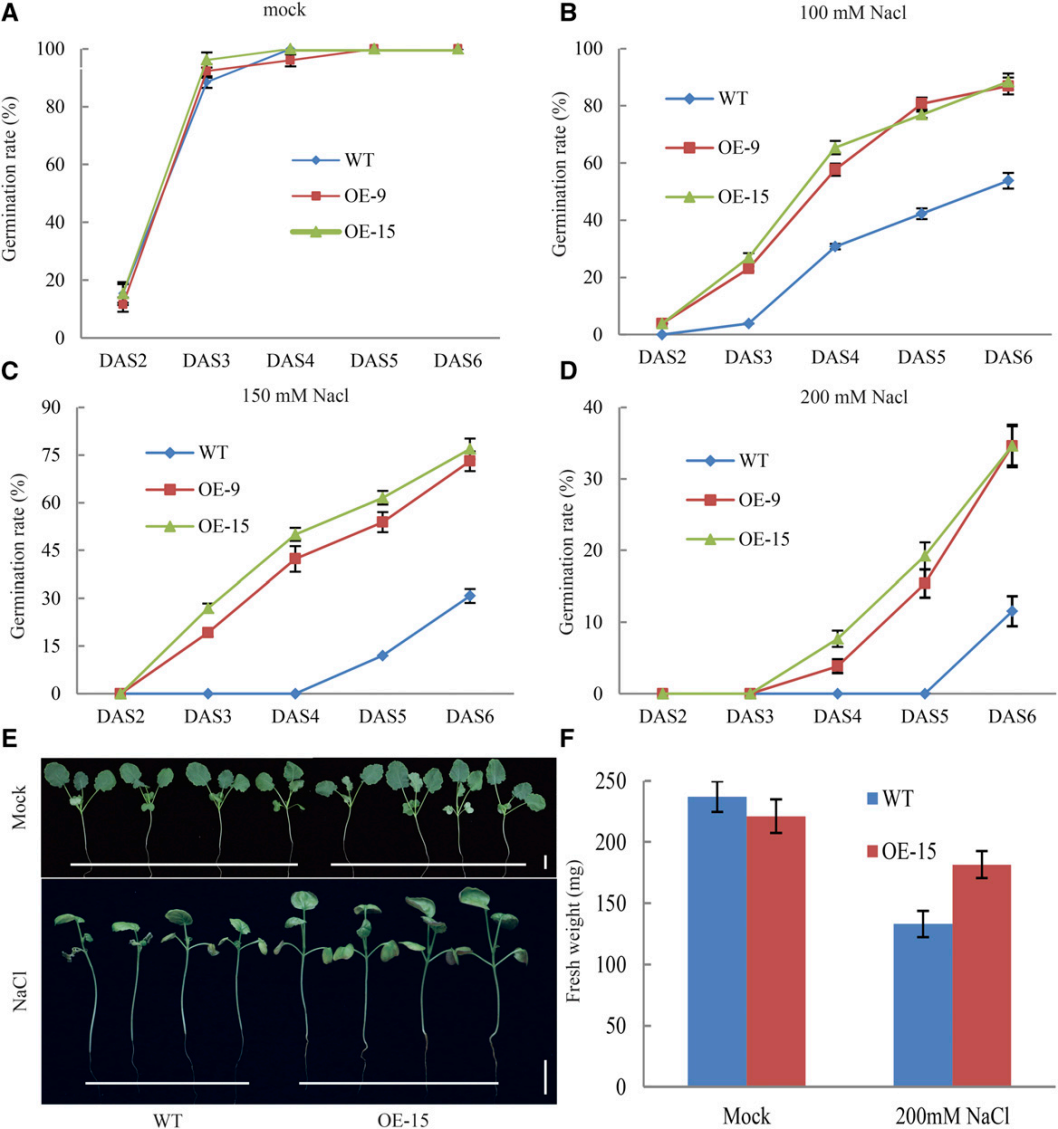
Germination and growth responses of transgenic plants to salt stress. sat stress. a-d, Seeds of WT and transgenic lines were germinated on plates containing different concentrations of 0-200mM NaCl separately. Germination rates were measured from DAS 2 to DAS 6; e-f, the roots of seedlings (DAS7) were immersed into a nutrient solution supplemented with 200 mM NaCl and fresh weight of seedlings was measured after one week. Bars = 2 cm in e.

Salt stress has both osmotic and ionic or ion toxicity effects on cells. For salt stress treatment, the seed germination rate between WT and OE lines was not different on regular MS medium without stress. Compared with the control condition, NaCl treatment resulted in a considerable delay in germination time and inhibition of seed germination. WT begun to germinate at DAS2 and the germination rate exceeded 90% under normal conditions. In contrast, seeds begin to germinate at DAS 3 with germination rate less than 10% under 100 mM NaCl treatment. When WT seeds were exposed to a 150 or 200 mM NaCl treatment, there was no germinate even at DAS 5 (Figure 5a). At DAS 4, only 30.77% of WT seed germinated, while the germination rate of *OE-15* reached to approximately 65.38% under 100 mM NaCl treatment (Figure 5b). When exposed to 150 mM NaCl treatment, approximately 42% of *OE-9* germinated, whereas WT seeds did not germinate at DAS 4. At DAS 6, the germination rate of the *OE* lines was greater than 70%, while that of WT was less than 30% (Figure 5c). When the NaCl concentration was increased to 200 mM, seed germination of WT and OE lines was more strongly suppressed. Initial germination time of *OE* lines was delayed by two days but the germination rate was still 24% higher than that of WT at DAS 6 (Figure 5d).

To study the effect of salt stress on seedling growth, the roots of seedlings (DAS7) were immersed into a nutrient solution supplemented with 200 mM NaCl for one weeks. High salt caused growth retardation of WT and transgenic plants. WT exhibited severe growth inhibition, including cotyledon necrosis and wilting at the junction of hypocotyl and radicle (Figure 5e). Fresh weight of WT decreased 44% whereas that of transgenic plants decreased by only 18% (Figure 5f). These results indicated that overexpression of *BnaAOX1b* positively regulated the seed tolerance to salt stress.

### Overexpression of Bna.AOX1b regulates sensitivity to ABA

**Figure 6:**
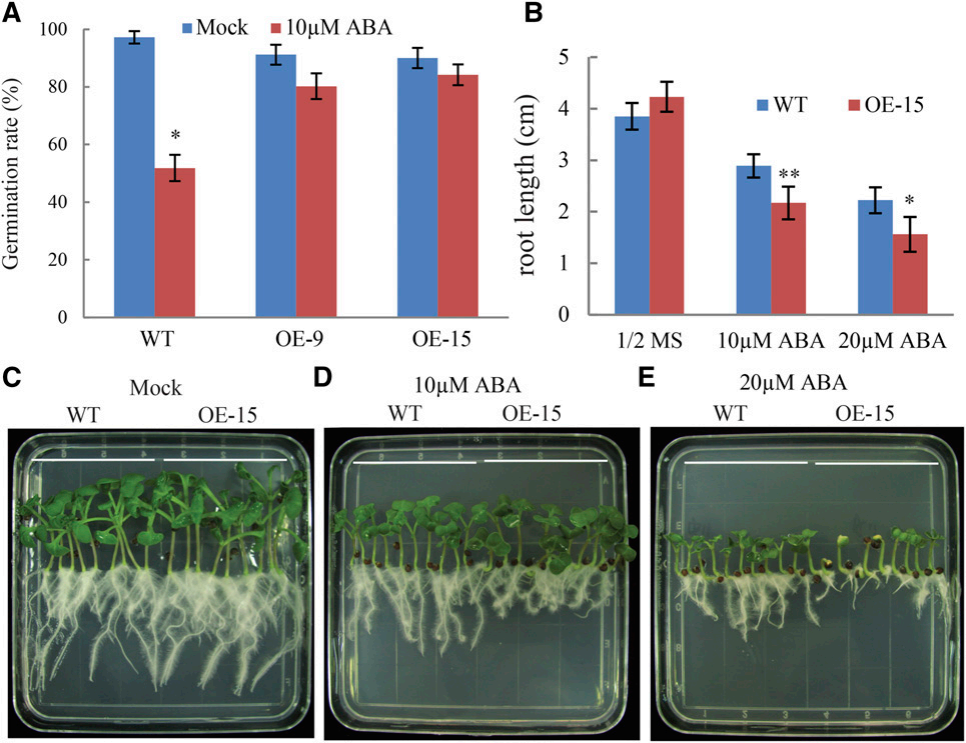
Germination and growth responses of transgenic plants to ABA. a, a, Naked seeds of the WT and transgenic lines were sown on plates containing 10μM ABA. Germination rates were measured at DAS 6. b-e, *BnaAOX1b* transgenic seedlings show increased sensitivity to ABA. The root length was measured at DAS 7. Vertical bars represent the SD of the means (n = 3, treatments with 30 seedlings).

ABA is an important hormone and plays an essential role in the response to salt stress. To determine whether *Bna*.*AOX1b* is involved in the ABA signaling pathway, the ABA sensitivity of the transgenic lines was tested during germination and seedling stages. Six days after 10 μM ABA treatment, seed germination rate of WT decreased by 39% whereas the germination rate of transgenic lines decreased by only 19% and 11%, respectively (Figure 6a). Overexpression of *BnaAOX1b* reduced sensitivity to ABA in terms of germination rate.

We further examined the response of seedling growth to ABA. When grown on 1/2 MS medium, *BnaAOX1b* transgenic plants grew at a similar rate as the wild type (Figure 6b-c). However, primary root growth of *OE-15* was more severely inhibited than WT plants after cultured on 1/2 MS medium with 10–20 μM ABA for seven days (Fig. b-e). When grown on 1/2 MS medium containing 10 μM ABA, there was no significant difference in the shoot elongation values between transgenic lines and WT (1.53 and 1.48 cm, respectively). In contrast, the root length of WT (2.88 cm) was significantly longer than that of transgenic lines (2.17 cm). Compared with the 10 μM ABA treatment, 20 μM ABA treatments resulted in greater inhibition of root elongation. The root length of the transgenic plants decreased to 1.56 cm, which was significantly shorter than that of WT (2.22 cm).

### Overexpression of Bna.AOX1b regulates genes related to ABA and osmotic stress in seed germination

**Figure 7:**
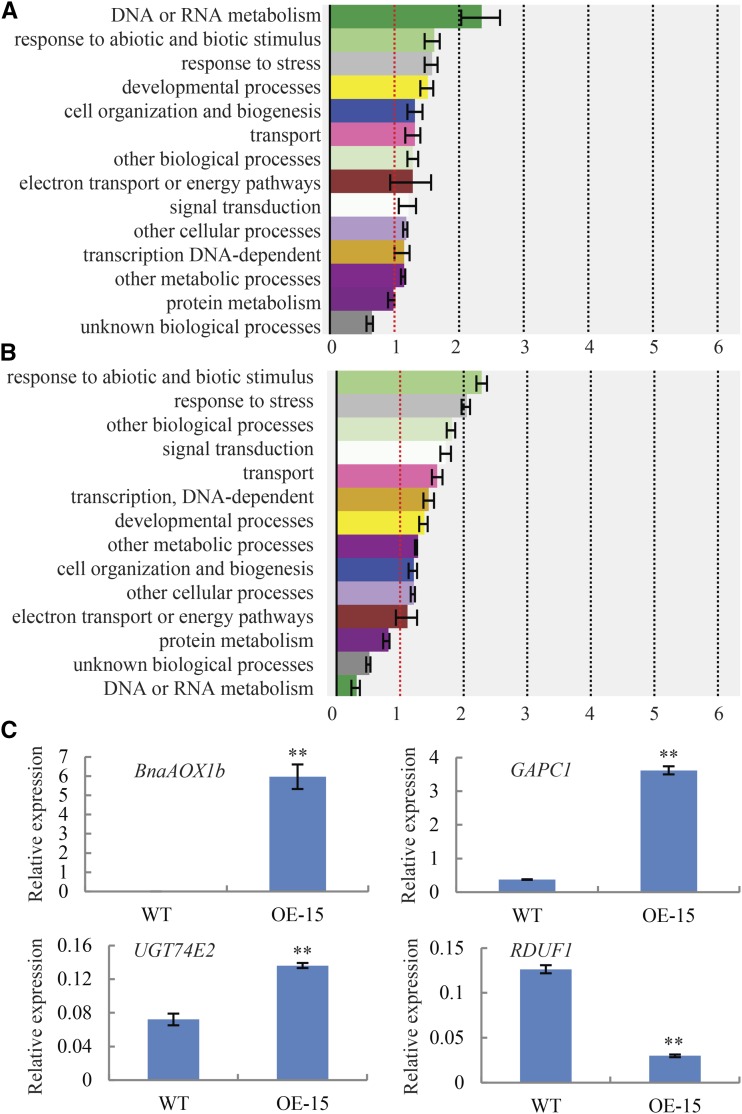
Analysis of differentially expressed genes in overexpressing *BnaAOX1b* transgenic plants. a, Up-expressed genes classified with GO; b, Down-regulated genes classified with GO; c, Quantitative PCR detected the expression level of candidate genes in *OE15* relative to WT (Log2).

To investigate the genome-wide effects on transcription resulting from *BnaAOX1b* overexpression, we compared the differentially expressed genes (DEGs) between transgenic line and WT. RNA from germinated seeds was chosen for gene expression analyses. Using the twofold change and the false discovery rate (FDR), 0.05 as the p-value cutoff for selecting the differentially expressed transcripts, several DEGs were identified. Genetic upregulation of *BnaAOX1b* expression level in rapeseed resulted in increased expression of 1501 genes and decreased expression of 1451 genes. To classify and analyze the genes affected by *BnaAOX1b*, the differentially expressed genes (DEGs) were submitted to an enrichment analyses. When classified with Gene Ontology (GO) terms, the gens response to stress or stimulus were both highly represented and significantly enriched in up-regulated and down-regulated genes (Figure 7a-b). AOX is the terminal oxidases of respiratory electron transport chain. The genes of electron transport or energy pathways showed the higher frequency of expression changes among the regulated genes (Figure 7a-b).

Corresponding to AOX function and the phenotypic variation of *BnaAOX1b* over-expression lines, DEGs in potential AOX-regulated pathways were analyzed. We focused on the genes that participate in osmotic stress or the response to ABA. The expression of 56 DEGs involved in response to ABA and 50 genes involved in osmotic stress were significantly changed (Table 2). These included *CSD1* (BnaA06g05150D), which functions in scavenging hydrogen peroxide or detoxifying superoxide radicals in plant cells. *CPK1* (BnaC05g36000D), which plays an important role in salt/drought-stress, had greater transcript abundance in transgenic plants compared with WT plants. In addition, 63 DEGs are responsible for response to oxidative stress and 23 genes are involved in response to ROS (Table 2). To ensure the reliability of our RNA-Seq data, we analyzed the expressions of several regulated genes in RNA-Seq data, including *BnaAOX1b*, *GAPC1*, *RDUF1* and so on. *RDUF1* exhibited down-regulation in *OE-15* where other detected genes exhibited up-regulation, consistent with our RNA-Seq results (Figure 7c).

**Table 2:** Go analysis of differentially expressed genes in WT and OE-15

Gene set name	GO ID	Gene number in overlap	Gene ID
response to ABA	0009737	56	BnaA02g07020D,BnaC05g42110D,BnaAnng12630D,BnaCnng06440D,BnaA04g05850D,BnaC05g47770D,BnaA05g33270D,BnaA03g37990D,BnaC04g56100D,BnaCnng28090D,BnaA09g27780D,BnaCnng78190D,BnaC06g28190D,BnaC03g42320D,BnaA04g01310D,BnaA06g17960D,BnaA01g02240D,BnaA07g26170D,BnaA05g32630D,BnaA06g31640D,BnaC06g09080D,BnaA10g07630D,BnaC04g40810D,BnaC08g01410D,BnaCnng37330D,BnaA08g28350D,BnaA05g32160D,BnaA09g14730D,BnaA10g07350D,BnaA04g29310D,BnaA04g01300D,BnaC07g07150D,BnaC06g37910D,BnaC01g09530D,BnaCnng06050D,BnaC03g44110D,BnaCnng29110D,BnaC07g06920D,BnaA05g30970D,BnaC01g03510D,BnaC07g11700D,BnaCnng27850D,BnaC06g40270D,BnaC09g30700D,BnaC07g25010D,BnaC05g36000D,BnaC08g46320D,BnaCnng37520D,BnaC05g01110D,BnaA05g11420D,BnaC03g21240D,BnaA10g20490D,BnaC05g21480D,BnaC05g47760D,BnaA03g08150D,BnaCnng06020D
response to osmotic stress	0006970	50	BnaA04g05850D,BnaA01g30570D,BnaA09g03360D,BnaA06g39830D,BnaAnng30680D,BnaC04g31100D,BnaC04g05620D,BnaAnng12720D,BnaC02g41600D,BnaA09g27780D,BnaCnng78190D,BnaA01g35180D,BnaC06g01320D,BnaA01g02240D,BnaA06g15800D,BnaC03g08320D,BnaC05g14070D,BnaCnng00290D,BnaAnng38290D,BnaC07g27220D,BnaC09g44630D,BnaC08g01410D,BnaA08g28350D,BnaAnng26260D,BnaA09g14730D,BnaCnng75020D,BnaC07g29830D,BnaAnng13220D,BnaA10g01360D,BnaC06g37910D,BnaA06g05150D,BnaC07g06920D,BnaC04g47470D,BnaC01g03510D,BnaC07g11700D,BnaC01g21880D,BnaC09g35160D,BnaA06g27890D,BnaA03g28780D,BnaA03g26140D,BnaC01g40530D,BnaC05g36000D,BnaC03g30880D,BnaC08g46320D,BnaC09g02710D,BnaA09g25940D,BnaA10g20610D,BnaC05g21480D,BnaA05g05760D,BnaA09g14280D
response to oxidative stress	0006979	63	BnaA09g53510D,BnaA05g33350D,BnaC05g42110D,BnaA04g04860D,BnaA03g22890D,BnaA01g30570D,BnaC03g28340D,BnaA03g02050D,BnaC04g05620D,BnaAnng12720D,BnaA02g22320D,BnaC03g78270D,BnaC04g20960D,BnaC04g56100D,BnaA09g02250D,BnaA09g27780D,BnaC02g02310D,BnaC05g45710D,BnaAnng12670D,BnaCnng00330D,BnaCnng67980D,BnaA09g43010D,BnaA09g05060D,BnaA03g17800D,BnaC09g44630D,BnaC08g01410D,BnaA05g31170D,BnaA08g28350D,BnaC02g15500D,BnaC03g50060D,BnaC04g49930D,BnaA10g25000D,BnaA08g00950D,BnaA07g14820D,BnaA04g04440D,BnaA06g05150D,BnaC04g47470D,BnaC03g26940D,BnaA05g27950D,BnaC07g11700D,BnaA08g24320D,BnaC09g35160D,BnaCnng06300D,BnaC07g43770D,BnaCnng29190D,BnaC09g24050D,BnaA04g25090D,BnaAnng16310D,BnaC08g46320D,BnaA01g03180D,BnaAnng16530D,BnaA05g11420D,BnaCnng18330D,BnaCnng05940D,BnaAnng01320D,BnaA01g06830D,BnaC09g49920D,BnaA10g20610D,BnaC05g21480D,BnaA05g05760D,BnaCnng06230D,BnaC09g16820D,BnaA09g22680D

## Discussion

In Arabidopsis, the AOX gene family contains five members, *AOX1a-1d* and *AOX2* whereas Legumes (*e.g.*, *Medicago sativa*, *C. arietinum*) contains one *AOX1* isoform and three *AOX2* isoforms (Sweetman *et al.* 2018). In rice and barley, AOX is encoded by four genes (rice:*OsAOX1a*, *1c*, *1d* and *1e*; barley: *HvAOX1a*, *1c*, *1d1* and *1d2*) representing four clades (Wanniarachchi *et al.* 2018). In this study, eight *AOX1* and two *AOX2* members were identified, which indicated that this gene family has expanded compared to that in Arabidopsis and rice, but no *AOX1c* type genes were found in rapeseed. CysI and CysII of AOX isoenzymes are both involved in 2-oxo acid activation and CysIII affected activity (Selinski *et al.* 2017). In rapeseed, most AOX isoforms possessed highly conserved CysI and CysII in the N-terminal domain of the protein, while AOX1b and two AOX1a loci possessed a third Cys residue (CysIII) near the catalytic di-iron center (Figure 1b).

Among the *AOX* genes, *Aox1a* and *Aox1d* are the most stress responsive genes following abiotic stress. *Aox1a* is the main AOX isoform and its transcript expression is relative abundant throughout Arabidopsis development stage. The expression of *Aox1b* is floral-specific *(**Wellmer et al.* 2004; Clifton *et al.* 2006; Hennig *et al.* 2004). The *Aox1d* transcript is predominately found in senescent leaves and during flowering, whereas *Aox2* expression is limited to the latter stages of silique maturation and in the germinating seed (Nakabayashi *et al.* 2005; Winter *et al.* 2007). The mRNA of rice *Aox1a* and *Aox1b* are mainly present in young roots and mature leaves. In legume species, *Aox2a* is strongly expressed in photosynthetic tissues, whereas *Aox1* is the most highly expressed in root (Sweetman *et al.* 2018). Differential activation of *AOX1a* and *AOX1b* at the transfer of young rice seedlings from 28° to 4° has been reported (Ito *et al.* 1997). Our results indicate that *Bna.AOX1b* is specially expressed in the ovule and displays differential expression between rapeseed cultivars which exhibit different salt resistance in seed germination. Differential expression of gene family members depends on the plant, tissues, growth, development and environment.

Seed germination and post-germination seedling growth of various plant species are arrested in response to unfavorable environmental conditions by signaling events involving the phytohormone ABA. ABA auxotrophs of many plant species exhibit enhanced germination potential and over accumulation of ABA enhances dormancy or delays germination (Okamoto *et al.* 2010; Salaita *et al.* 2005). Our study consistently showed that overexpression of *BnaAOX1b* reduces ABA sensitivity and improves the germination rate under salt stress in terms of seed germination. The genes that participate in response to ABA were altered in *BnaAOX1b* transgenic lines, including *ABI4*, *SALT TOLERANCE ZINC FINGER* (*ZAT10/STZ*), *UGT74E2*, *DREB19*, *Ca^2+^ binding protein 1* (*CP1*), among others. In addition, ABA mediates some aspects of the physiological responses to environmental stress. Stress signals are converted to ABA, and this triggers the activation of a number of plant physiological and developmental processes, thereby inducing adaptation to the stress (Ton *et al.* 2009; Finkelstein *et al.* 2002). Transgenic plants of *BnaAOX1b* exhibit hypersensitivity to ABA with enhanced salt tolerance during post-germination. It is suggested that the sensitivity to ABA facilitates a rapid response to stress in transgenic plants. Previous studies report that enhanced tolerance to water deficit or salt stress accompanied by hypersensitivity to ABA treatment during early seedling development (Hu *et al.* 2006; Ko *et al.* 2006; Li *et al.* 2017).

According to Food and Agriculture Organization (2008), 7% of the world’s land has been salinized, and this percentage is still increasing. High salt concentration induces water deficit, ionic toxicity, nutrient imbalance, and oxidative stress, which affects plant growth and development. Salt stress greatly reduces crop productivity and salinization of agricultural land has been a major problem worldwide for decades. *AOX* has been proposed as a functional marker for breeding stress tolerant plant varieties (Arnholdt-Schmitt *et al.* 2006). *BnaAOX1b* showed differential expression among the rapeseed cultivars zy036 and 51070 with different resistance. Overexpression of *BnaAOX1b* substantially increases the germination rate compared to the WT under salt stress, suggesting that *BnaAOX1b* is a positive regulator which confers tolerance to salt stress.
